# Effects of Silymarin-Loaded Nanoparticles on HT-29 Human Colon Cancer Cells

**DOI:** 10.3390/medicina54010001

**Published:** 2018-03-09

**Authors:** Maryam Mombeini, Ghasem Saki, Layasadat Khorsandi, Neda Bavarsad

**Affiliations:** 1 Cell & Molecular Research Center, Ahvaz Jundishapur University of Medical Sciences, Ahvaz 61335, Iran; maryammombeini71@gmail.com (M.M.); ghasemsaki@yahoo.com (G.S.); 2 Department of Anatomical Sciences, Faculty of Medicine, Ahvaz Jundishapur University of Medical Sciences, Ahvaz 61335, Iran; 3 Nanotechnology Research Center, Ahvaz Jundishapur University of Medical Sciences, Ahvaz 61335, Iran; nbavarsad@gmail.com; 4 Department of Pharmaceutics, School of Pharmacy, Ahvaz Jundishapur University of Medical Sciences, Ahvaz 61335, Iran

**Keywords:** silymarin, micelle, colon cancer, nanodrug

## Abstract

*Background and objective*: Previous studies have demonstrated the anti-cancer effects of silymarin (SLM). However, the low bioavailability of SLM has restricted its use. This study investigated the toxic effect of nanostructured SLM encapsulated in micelles (Nano-SLM) on the growth of the HT-29 human colon cancer cell line. *Materials and methods*: HT-29 cells were treated with 25 μM/mL of SLM or Nano-SLM for 48 h. MTT and colony formation assays were used to assess the cytotoxicity and proliferation of HT-29 cells, respectively. The cells were stained with annexin V/PI for assessment of apoptosis. *Results*: MTT assays revealed that Nano-SLM treatment was able to exert a more pronounced toxic effect on the HT-29 cells as compared to free SLM treatment (*p* < 0.01). In the Nano-SLM-treated cells, colony numbers were significantly reduced in comparison to the free SLM-treated cells (*p* < 0.01). Apoptotic and necrotic indexes of Nano-SLM-treated HT-29 cells were also significantly increased in comparison to those of the free SLM-treated cells (*p* < 0.01). The viability, proliferation and apoptosis of healthy cells (NIH-3T3 cells) were not changed in response to Nano-SLM or SLM. *Conclusions*: Our results indicate that Nano-SLM enhances the anti-cancer effects of SLM against human colon cancer cells.

## 1. Introduction

Colon cancer is one of the most diagnosed cancers. Chemotherapy and radiotherapy have many untoward side effects on healthy cells and the cancer cells are often drug resistant [1,2]. Colon cancer has strong associations with dietary factors. Plants contain a wide variety of phytochemicals, some of which are able to protect healthy cells from various processes that can induce cancer.

Silymarin (SLM), extracted from *Silybum marianum* (Milk thistle) fruit, is composed of one flavonoid (taxifolin) and seven flavolignans including silychristin A, silychristin B, silydianin, silybin B, silybin A, isosilybin A and isosilybin B. Among these, Silybin (also named silibinin) is the most abundant flavolignan extracted from SLM (50–70%) [3].

Recent in vivo and in vitro studies have demonstrated the remarkable anti-cancer effects of SLM and its derivatives on several cancers, such as lung, prostate, bladder and colon cancers [4]. However, the low bioavailability of SLM restricts its therapeutic efficacy [5]. It has been reported that the encapsulation of SLM in polymer nanoparticles, liposomes, micelles and solid lipid nanoparticles improves its solubility and bioavailibity [6,7,8,9,10]. Liposomes are used to deliver small lipophilic and hydrophilic agents, large proteins and nucleic acids. Liposomes are a closed lipid bilayer with an aqueous internal compartment and are able to increase the therapeutic safety and activity of drugs [7,11,12,13,14,15,16]. Micelles are composed of lipid monolayers separated by a fatty acid core [17]. Micelles possess a size range of 5 to 20 nm; they are smaller than liposomes [18]. Elmowafy et al. (2013) reported that SLM-loaded liposome was significantly better than free SLM and the liposome significantly increased the cellular uptake of SLM [19]. In a previous study, the absorption of SLM micelles at different parts of the intestine was significantly higher than the free SLM in rats [5]. In the study of Li et al. (2009), micelles significantly elevated the amount of silybin in liver tissue [10]. The objective of this project was to compare the cytotoxic effects of SLM and nanostructured SLM (Nano-SLM) on HT-29, a human colon cancer cell line.

## 2. Materials and Methods

### 2.1. Preparation of Nano-SLM

Nano-SLM was prepared by a lipid-thin layer of hydration film [20]. Briefly, SLM (10 mg) and soy phosphatidylcholine and cholesterol in a molar ratio of 6:1 were dissolved in a chloroform–methanol solution (9:1 *v/v*: 9 mL chloroform and 1 mL methanol). The solvent was evaporated at 40 °C under reduced pressure (120 rpm) using a rotary evaporator to develop a thin lipid film. This film was maintained at 40 °C overnight to completely withdraw the solvent. The hydrated film with 4 mL phosphate buffer (pH: 7.4) was sonicated three times at 50 Hz in a bath-sonicator and homogenized at 20,000 psi for five cycles. Unloaded SLM was then separated by centrifuging at 13,000 rpm at 4 °C. SLM, as a mixture of flavonolignans from the fruit of *Silybum marianum*, was purchased from Sigma (Cat number: S0292).

### 2.2. Characterization of Nano-SLM

Mean particle size was determined using a DLS particle size analyzer (Qudix, Scatterscope I, Seoul, South Korea) after suitable re-dispersion in phosphate buffer. The average size distribution of Nano-SLM was determined by a Zetasizer-Nano-ZSP (Malvern, UK) after being diluted 10 times with double-distilled water at room temperature. In addition, micelles were suspended in phosphate buffer and particle shape was visualized by transmission electron microscopy (TEM).

### 2.3. Drug Entrapment Efficiency

The prepared Nano-SLM was centrifuged at 13,000 rpm for 5 min to separate non-entrapped SLM. Supernatant was analyzed for testing SLM. The encapsulation efficiency was calculated using the following formula [21].

% Encapsulation efficiency = (Total drug − amount of drug in supernatant/Total drug) × 100(1)

### 2.4. In Vitro Drug Release

Nano-micelle samples enclosed in dialysis bags (cellulose membrane MW cut-off 12 KDa, Sigma) were immersed in phosphate buffer (PH: 7.4) at 25 ± 1 °C and were stirred at 100 rpm. At predetermined time intervals, samples were withdrawn and analyzed for SLM spectrophotometrically at 327 nm. The following formula was used to calculate the percentage of drug released [21].

%Drug released = (Amount of drug release/amount of drug in micelle) × 100(2)

### 2.5. Experimental Design

The human HT-29 cell line and a mouse healthy fibroblast cell line (NIH-3T3) were obtained from Pasteur Institute of Iran. The cell lines were cultured in DMEM equilibrated with 5% CO^2^ atmosphere at 37 °C. Untreated cells constituted the control group. Experimental groups were treated by 25 μM/mL of SLM, 25 μM/mL of Nano-SLM and 25 μM/mL of blank micelles, respectively, for 48 h. The dose of SLM and Nano-SLM was selected based on our pilot study.

### 2.6. Cell Viability

The MTT (3-(4, 5-Dimethylthiazol-2-yl)-2,5-diphenyltetrazolium-bromide) assay (the best known method for determining mitochondrial dehydrogenase activities in the living cells) was used to compare the effect of Nano-SLM with SLM on cell viability as previously described. Briefly, HT-29 (10,000 cells/well) cells were maintained with culture media for 48 h in 96-well plates. MTT (0.5 mg/mL) solution was then added to each well, and the cells were further incubated for 4 h at 37 °C. Supernatants were then removed, and 100 µL of Dimethyl sulfoxide (DMSO) was added to each well to dissolve the formazan product. Absorbance at 540 nm was measured using a microplate reader (BioRad, Hercules, CA, USA). The results of the MTT assay were expressed as the percentage of corresponding average values in control cells [22].

### 2.7. Clonogenicity Assay

For the assessment of colony formation, 500 cells were seeded into 6-well culture dishes and exposed to SLM or Nano-SLM in serum-free medium for 48 h. The cells were washed and further incubated with complete medium for 10 days. The cells were stained with 0.1% crystal violet in PBS, and the colonies were counted under a light microscope [23].

### 2.8. Annexin V-FITC/Propidium Iodide Apoptosis Assay

HT-29 cells (10^5^) were treated with SLM or Nano-SLM for 48 h as experimental groups. Normal, apoptotic and necrotic cells were determined using an Annexin V-FITC/propidium iodide assay kit (Invitrogen, V13242, Molecular Probes/Invitrogen, Carlsbad, CA, USA) as previously described. The samples were analyzed with a Flow cytometer (Becton Dickinson, Franklin Lakes, NJ, USA). The different labeling patterns in the Annexin V/PI analysis identified the different cell populations where FITC-negative and PI-negative were designated as viable cells; FITC-positive and PI-negative as early apoptotic cells; FITC-positive and PI-positive as late apoptotic cells and FITC-negative and PI-positive as necrotic cells. The data analysis was performed using WinMDI 2.9 software (J. Trotter, The Scripps Research Institute, La Jolla, CA, USA) [23].

### 2.9. Statistical Analysis

The data were compared by one-way analysis of variance (ANOVA) (SPSS Inc., Chicago, IL, USA—SPSS version 19.0). This was followed by a post hoc pair-wise comparison using the Bonferroni t-procedure. *p* < 0.05 was considered significant.

## 3. Results

### 3.1. Characterization of Nano-SLM

**Figure 1:**
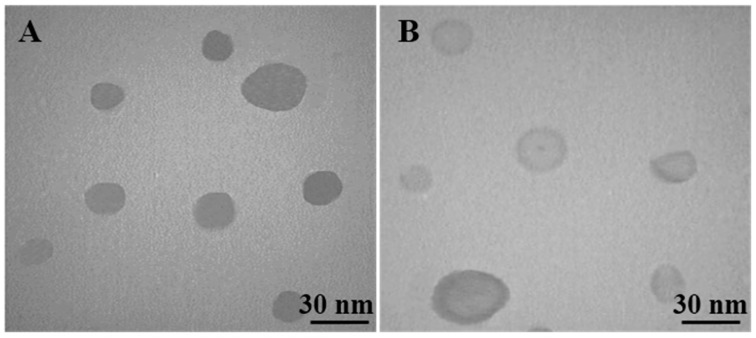
TEM micrograph of blank nano-micelles (**A**) and Nano-SLM nanoparticles (**B**).

The particle size distribution showed a range of 20 nm to 30 nm, with the mean particle size being nearly 26.5 nm. The zeta potential of Nano-SLM indicated that it exhibited a very good stability for loading free SLM. The morphology of Nano-SLM with TEM is shown in Figure 1. The lipid layer of the micelles appeared as dark rings around the internal aqueous media. The TEM images showed that the targeted micelles were of a discrete, uniform and regular round shape. The sizes of micelles determined from TEM measurements were 26.1 ± 4.3 nm. The sizes obtained from the TEM measurements are in good accordance with the results obtained from the particle size measurements by dynamic light scattering. These data demonstrate that SLM-loaded micelles can be a stable drug carrier with narrow particle size, steady zeta potential, and closely graded shape.

**Figure 2:**
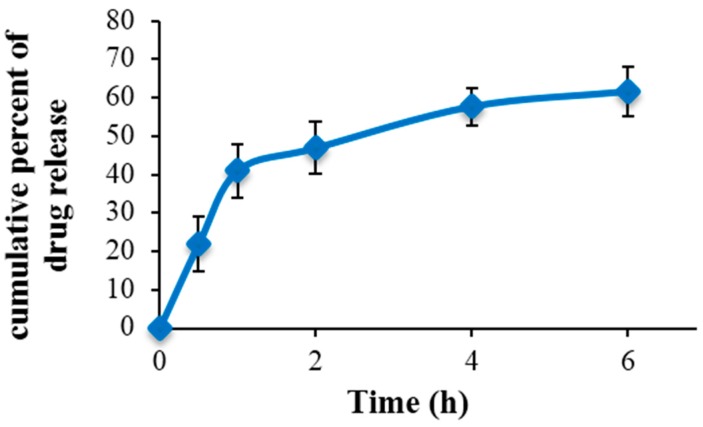
In vitro cumulative percent drug release vs. in time. Data expressed as mean ± SD (*n* = 6).

The encapsulation efficiency of Nano-SLM was 99.48%. The release profile in vitro showed an initial burst release for 0.5 to 6 h and then exhibited a slow release of SLM (Figure 2). Furthermore, the drug release rate data indicated that the slow release of Nano-SLM had lasted nearly 48 h. These findings illustrated that Nano-SLM could indeed provide a slow release performance for SLM and it has great potential applicability as an SLM carrier, enabling continuous provision during the treatment. In addition, the prepared Nano-SLM was completely dispersed in aqueous media with no aggregate as opposed to free SLM which exhibits poor aqueous solubility. These results are summarized in Table 1.

### 3.2. Cell Viability and Proliferation

**Figure 3:**
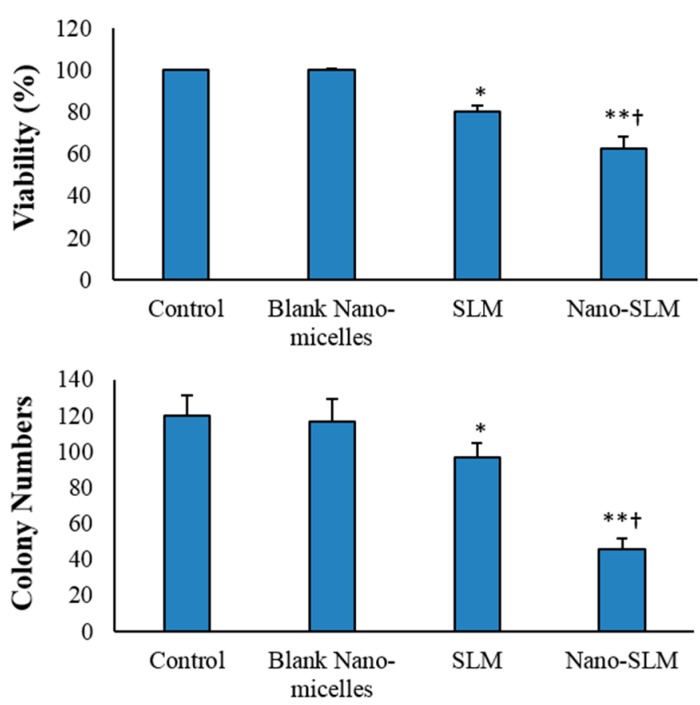
Percentage of cell viability and colony numbers of HT-29 cells in the control and experimental groups. All assays were performed in triplicate, and the mean ± standard deviations are shown. * *p* < 0.01, ** *p* < 0.001, † *p* < 0.001; * and † symbols indicate comparison to the control and SLM groups, respectively.

**Figure 4:**
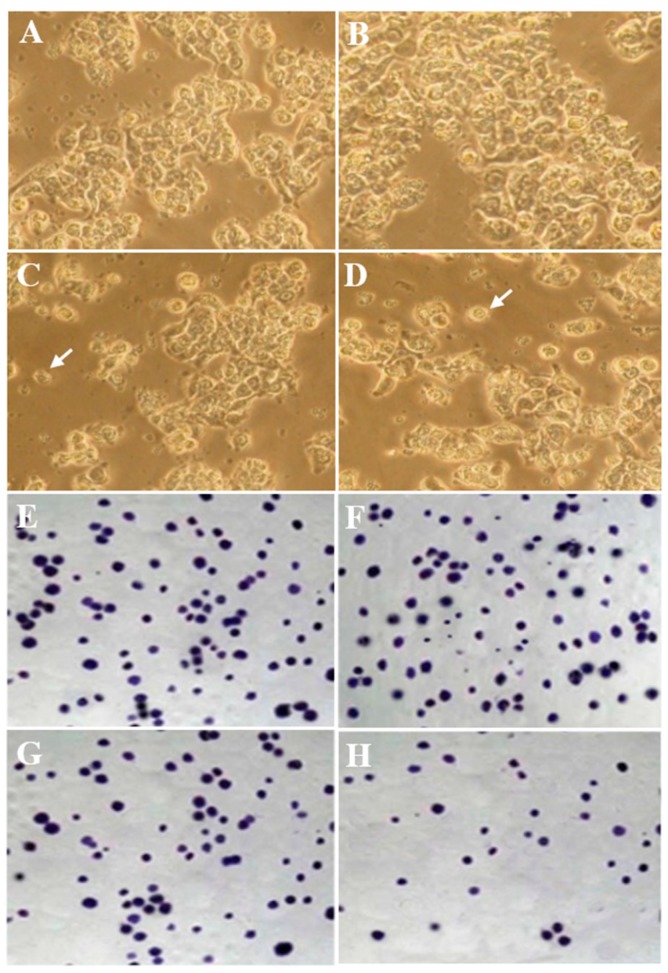
Morphology (**A**–**D**) and clonogenicity (**E**–**H**) of HT-29 cells. A and E, control untreated cells; (**B**,**F**), blank nano-micelles treatment; (**C**,**G**), SLM-treated cells; (**D**,**H**), Nano-SLM-treated cells; Arrows indicate apoptosis. All magnifications are × 400.

Free SLM significantly decreased the viability percentage of HT-29 cells (*p* < 0.05). In the Nano-SLM-treated cells, the viability of HT-29 cells was significantly decreased compared to that of the free SLM-treated cells (*p* < 0.01). Free SLM significantly decreased the colony numbers of HT-29 cells (*p* < 0.05). In the Nano-SLM-treated cells, the colony formation of HT-29 cells was significantly decreased in comparison to that of the free SLM group (*p* < 0.01). In the blank micelles-treated cells, the percentages of cell viability and colony numbers were similar to those of the control (Figure 3 and Figure 4). The proliferation and viability of NIH-3T3 cells were not significantly affected by SLM or Nano-SLM (Results not shown).

### 3.3. Morphology Evaluation

In the control group, a small number of HT-29 cells showed round morphology. In the blank micelles-treated cells, the morphology was similar to that of the control untreated cells. The free SLM-treated HT-29 cells exhibited apoptotic morphology, including round shape, cell membrane blebbing and nucleus condensation. These morphological features were considerably more prominent in the Nano-SLM-treated cells (Figure 4). The morphology of the NIH-3T3 cells was not affected by SLM or Nano-SLM treatment (Results not shown).

### 3.4. Annexin V-FITC/Propidium Iodide Apoptosis Assay

**Figure 5:**
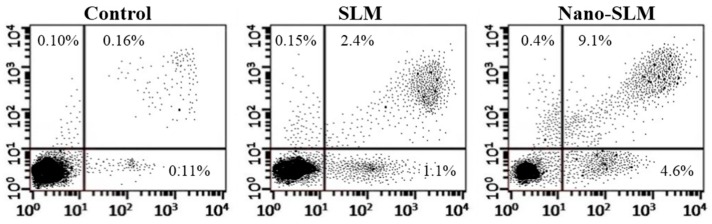
Flow cytometry of Annexin/PI staining in the control and experimental groups. The lower left quadrant (Annexin V-FITC−/PI−): live cells; the lower right quadrant (Annexin V-FITC+/PI−): early-stage apoptotic cells; the upper right quadrant (Annexin V-FITC+/PI+): late-stage apoptotic cells; the upper left quadrant (Annexin V-FITC−/PI+): necrotic cells.

**Figure 6:**
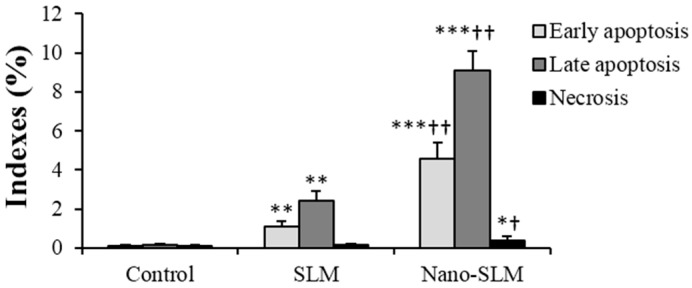
Indexes of necrosis and apoptosis of HT-29 cells in the control and experimental groups. All assays were performed in triplicate, and the mean ± standard deviations are shown. * *p* < 0.05, ** *p* < 0.001, *** *p* < 0.001, † *p* < 0.01, †† *p* < 0.001; * and † symbols indicate comparison to the control and SLM groups, respectively.

In the SLM-treated cells, early (FITC+/PI−) and late apoptosis (FITC+/PI+) were significantly more common than in the control untreated cells. In the Nano-SLM-treated cells, the percentage of early and late apoptosis was significantly increased in comparison to the free SLM-treated cells (*p* < 0.01). In the SLM-treated cells, the necrotic index (FITC−/PI+) was significantly greater than that of the control. In the Nano-SLM-treated cells, the percentage of necrotic cells was significantly increased in comparison to the free SLM group (*p* < 0.01). In the blank micelles-treated cells, apoptotic and necrotic indexes were similar to that of the control (Figure 5 and Figure 6). The apoptotic and necrotic indexes of NIH-3T3 cells were not affected by SLM or Nano-SLM treatment (Results not shown).

## 4. Discussion

This study has demonstrated that nanostructuring SLM encapsulated in micelles effectively enhances its cytotoxicity effects. MTT assessments showed that Nano-SLM treatment significantly reduced the viability of HT-29 cells in comparison to free SLM treatment. The enhanced cytotoxicity of SLM encapsulated in micelles may relate to its increased penetration into the HT-29 cells. Elmowafi et al. showed that SLM encapsulated in liposomes significantly enhanced the anti-cancer activities of SLM, which was associated with its enhanced solubility and stability, and increased SLM content in HepG2 cancer cells [19]. In the study of Yazdi-Rouholamini (2010), Nano-silibinin (also named silybin) was more effective than free silibinin in the T47D breast cancer cell line [24].

Wang et al. (2017) demonstrated that small micelles (less than 30 nm) significantly improve the therapeutic efficacy of drugs in comparison to large micelles (100 to 160 nm) in tumors [25]. Ong et al. (2016) showed that high drug encapsulation efficiency and small liposome size could enhance the oral bioavailability of griseofulvin-loaded liposomes [26]. As shown in the results, the mean particle size of Nano-SLM was less than 30 nm—a little larger than that of blank micelles. The slight increase in particle size of Nano-SLM might result from the entrapment of free SLM into micelles. Sui et al. (2010) showed that SLM-loaded micelles were bigger than blank micelles [27].

Nano-SLM effectively inhibited the proliferation of HT-29 cells. In a previous study, SLM induced cell cycle arrest and apoptosis in ovarian cancer cells [28].

To study whether the cytotoxicity effects of Nano-SLM on HT-29 cells were associated with the induction of apoptosis, we used the Annexin V/PI method and morphology assessments. Nano-SLM effectively induced late and early apoptosis in HT-29 cells. Nano-SLM also increased the necroptotic index in the HT-29 cells. The significant percentage of cell death detected by the Annexin V method was consistent with that detected by MTT and clonogenicity assessments. Snima et al. (2014) demonstrated that SLM encapsulated in poly (d,l-lactic-*co*-glycolic acid) nanoparticles can decrease cell viability and induce apoptosis in prostate cancer cell lines [6]. In another study, SLM inhibited proliferation and induced apoptosis in hepatic cancer cells [29].

Typical Nano-SLM-induced apoptosis features include cell shrinkage, membrane blebbing, condensed contents, and a rounded body in HT-29 cells. These morphological changes confirmed the results of annexin V/PI and were consistent with the viability percentage and colony numbers in the control and experimental groups.

In our study, SLM also decreased cell viability and proliferation, and increased apoptosis in HT-29 cells. Despite the low bioavailability of SLM, its therapeutic efficacy against various cancer cells has been documented [4,23]. Among several flavonoids and flavolignans of SLM, the anti-cancer effects of taxifolin and silybin (also named silibinin) have been demonstrated.

Lee et al. (2013) reported that silybin inhibited the growth of melanoma cells [30]. In the study of Imai-Sumida et al. (2017), silibinin significantly suppressed proliferation, migration and invasion, and induced apoptosis in T24 and UM-UC-3 human bladder cancer cells [31]. Li et al. (2017) showed that silibinin induced apoptosis in gastric cancer BGC823 cells [32]. Silibinin induced apoptosis in human choriocarcinoma cells [33]. The anti-cancer effects of SLM and silibinin on salivary gland cancer have also been reported by Choi et al. (2017) [34]. Chen et al. (2018) showed that taxifolin suppressed the proliferation of human osteosarcoma cells [35]. Alzaharna et al. (2017) demonstrated that taxifolin induced apoptosis and autophagy cell death in HeLa cells [36]. The anti-cancer effects of taxifolin on experimental colon carcinogenesis have also been documented by Manigandan et al. (2015) [37].

Interestingly, the viability, proliferation and apoptotic indexes in healthy cells were not affected by Nano-SLM. Thus, Nano-SLM has great potential as an adjuvant therapy for clinical application in colon cancer.

## 5. Conclusions

In summary, the encapsulation of SLM in micelles can effectively enhance its anti-cancer effects by activation of apoptosis in HT-29 cells. Future studies are needed to increase our knowledge about cell death signaling pathways in Nano-SLM-treated cancer cells.

## Figures and Tables

**Table 1:** Characteristics of the formulation of silymarin (SLM)/Blank micelles.

Groups	Particle Size	PDI	Zeta Potential
nm		Mv	
Nano-SLM	26.5 ± 4.3	0.53 ± 0.03	−23.5 ± 0.6
Blank micelles	23.4 ± 3.9	0.56 ± 0.04	−23.6 ± 0.5

Results are given as mean  ±  SD (*n* = 3). SD: standard deviation, PDI: polydispersity index.

## References

[1] Haggar F.A., Boushey R.P. (2009). Colorectal cancer epidemiology: Incidence, mortality, survival, and risk factors. Clin. Colon Rectal Surg..

[2] Hayat M.J., Howlader N., Reichman M.E., Edwards B.K. (2007). Cancer statistics, trends, and multiple primary cancer analyses from the Surveillance, Epidemiology, and End Results (SEER) program. Oncologist.

[3] Vargas-Mendoza N., Madrigal-Santillán E., Morales-González A., Esquivel-Soto J., Esquivel-Chirino C., García-Luna M., González-Rubio Y., Morales-González J.A. (2014). Hepatoprotective effect of silymarin. World J. Hepatol..

[4] Agarwal C., Wadhwa R., Deep G., Biedermann D., Gažák R., Křen V., Agarwal R. (2013). Anti-cancer efficacy of Silybin derivatives—A structure-activity relationship. PLoS ONE.

[5] Javed S., Kohli K., Ali M. (2011). Reassessing bioavailability of silymarin. Altern. Med. Rev..

[6] Snima K.S., Arunkumar P., Jayakumar R., Lakshmanan V. (2014). Silymarin encapsulated poly (d,l-lactic-*co*-glycolic acid) nanoparticles: A prospective candidate for prostate cancer therapy. J. Biomed. Nanotechnol..

[7] El-Samaligy M.S., Afifi N.N., Mahmoud E.A. (2006). Evaluation of hybrid liposomes-encapsulated silymarin regarding physical stability and in vivo performance. Int. J. Pharm..

[8] Yang G., Zhao Y., Zhang Y., Dang B., Liu Y., Feng N. (2015). Enhanced oral bioavailability of silymarin using liposomes containing a bile salt: Preparation by supercritical fluid technology and evaluation in vitro and in vivo. Int. J. Nanomed..

[9] Yang G., Zhao Y., Feng N., Zhang Y., Liu Y., Dang B. (2015). Improved dissolution and bioavailability of silymarin delivered by a solid dispersion prepared using supercritical fluids. Asian J. Pharm. Sci..

[10] Li X., Huang Y., Chen X., Zhou Y., Zhang Y., Li P., Liu Y., Sun Y., Zhao J., Wang F. (2009). Selfassembly and characterization of Pluronic P105 micelles for liver-targeted delivery of silybin. J. Drug Target..

[11] Xu X., Khan M.A., Burgessa D.J. (2012). A two-stage reverse dialysis in vitro dissolution testing method for passive targeted liposomes. Int. J. Pharm..

[12] Pandey H., Rani R., Agarwal V. (2016). Liposome and Their Applications in Cancer Therapy. Braz. Arch. Boil. Technol..

[13] Paolino D., Cosco D., Gaspari M., Celano M., Wolfram J., Voce P., Puxeddu E., Filetti S., Celia C., Ferrari M. (2014). Targeting the thyroid gland with thyroid-stimulating hormone (TSH)-nanoliposomes. Biomaterials.

[14] Brown B.S., Patanam T., Mobli K., Celia C., Zage P.E., Bean A.J., Tasciotti E. (2014). Etoposide-loaded immunoliposomes as active targeting agents for GD2-positive malignancies. Cancer Biol. Ther..

[15] Celia C., Ferrati S., Bansal S., van de Ven A.L., Ruozi B., Zabre E., Hosali S., Paolino D., Sarpietro M.G., Fine D. (2014). Sustained zero-order release of intact ultra-stable drug-loaded liposomes from an implantable nanochannel delivery system. Adv. Healthc. Mater..

[16] Paolino D., Cosco D., Racanicchi L., Trapasso E., Celia C., Iannone M., Puxeddu E., Costante G., Filetti S., Russo D. (2010). Gemcitabine-loaded PEGylated unilamellar liposomes vs. GEMZAR: Biodistribution, pharmacokinetic features and in vivo antitumor activity. J. Control Release.

[17] Akbarzadeh A., Rezaei-Sadabady R., Davaran S., Joo S.W., Zarghami N., Hanifehpour Y., Samiei M., Kouhi M., Nejati-Koshki K. (2013). Liposome: Classification, preparation, and applications. Nanoscale Res. Lett..

[18] Florence A.T., Attwood D. (2006). Physicochemical Principles of Pharmacy.

[19] Elmowafy M., Viitala T., Ibrahim H.M., Abu-Elyazid S.K., Samy A., Kassem A., Yliperttula M. (2013). Silymarin loaded liposomes for hepatic targeting: In vitro evaluation and HepG2 drug uptake. Eur. Pharm. Sci..

[20] Arumugam K., Subramanian G.S., Mallayasamy S.R., Averineni R.K., Reddy M.S., Udupa N. (2008). A study of rivastigmine liposomes for delivery into the brain through intranasal route. Acta Pharm..

[21] Shivhare U., Ambulkar D., Mathur V., Bhusari K., Godbole M. (2009). Formulation and evaluation of pentoxifylline liposome formulation. Dig. J. Nanomater. Biostruct..

[22] Soleimani M., Khorsandi L., Atashi A., Nejaddehbashi F. (2014). Chondrogenic differentiation of human umbilical cord blood-derived unrestricted somatic stem cells on a 3D beta-tricalcium phosphate-alginate-gelatin scaffold. Cell J..

[23] Khorsandi L., Saki G., Bavarsad N., Mombeini M. (2017). Silymarin induces a multi-targeted cell death process in the human colon cancer cell line HT-29. Biomed. Pharmacother..

[24] Yazdi Rouholamini S.E., Moghassemi S., Maharat Z., Hakamivala A., Kashanian S., Omidfar K. (2017). Effect of silibinin-loaded nano-niosomal coated with trimethyl chitosan on miRNAs expression in 2D and 3D models of T47D breast cancer cell line. Artif. Cells Nanomed. Biotechnol..

[25] Wang J., Mao W., Lock L.L., Tang J., Sui M., Sun W., Cui H., Xu D., Shen Y. (2015). The Role of Micelle Size in Tumor Accumulation, Penetration, and Treatment. ACS Nano.

[26] Ong S.G., Ming L.C., Lee K.S., Yuen K.H. (2016). Influence of the encapsulation efficiency and size of liposome on the oral bioavailability of griseofulvin-loaded liposomes. Pharmaceutics.

[27] Sui W., Yin C., Kong X. (2010). Micellar solubilization and in vitro release of silymarin in the self-aggregates of an amphiphilic derivative of chitosan polymers and organic chemistry. Macromol. Symp..

[28] Fan L., Ma Y., Liu Y., Zheng D., Huang G. (2014). Silymarin induces cell cycle arrest and apoptosis in ovarian cancer cells. Eur. J. Pharmacol..

[29] Ramakrishnan G., Lo Muzio L., Elinos-Báez C.M., Jagan S., Augustine T.A., Kamaraj S., Anandakumar P., Devaki T. (2009). Silymarin inhibited proliferation and induced apoptosis in hepatic cancer cells. Cell Prolif..

[30] Lee M.H., Huang Z., Kim D.J., Kim S.H., Kim M.O., Lee S.Y., Xie H., Park S.J., Kim J.Y., Kundu J.K. (2013). Direct targeting of MEK1/2 and RSK2 by silybin induces cell-cycle arrest and inhibits melanoma cell growth. Cancer Prev. Res. (Phila).

[31] Imai-Sumida M., Chiyomaru T., Majid S., Saini S., Nip H., Dahiya R., Tanaka Y., Yamamura S. (2017). Silibinin suppresses bladder cancer through down-regulation of actin cytoskeleton and PI3K/Akt signaling pathways. Oncotarget.

[32] Li R., Yu J., Wang C. (2017). Silibinin promotes the apoptosis of gastric cancer BGC823 cells through caspase pathway. J. BUON.

[33] Ham J., Lim W., Bazer F.W., Song G. (2018). Silibinin stimluates apoptosis by inducing generation of ROS and ER stress in human choriocarcinoma cells. J. Cell. Physiol..

[34] Choi E.S., Oh S., Jang B., Yu H.J., Shin J.A., Cho N.P., Yang I.H., Won D.H., Kwon H.J., Hong S.D. (2017). Silymarin and its active component silibinin act as novel therapeutic alternatives for salivary gland cancer by targeting the ERK1/2-Bim signaling cascade. Cell. Oncol. (Dordr.).

[35] Chen X., Gu N., Xue C., Li B.R. (2018). Plant flavonoid taxifolin inhibits the growth, migration and invasion of human osteosarcoma cells. Mol. Med. Rep..

[36] Alzaharna M., Alqouqa I., Cheung H.Y. (2017). Taxifolin synergizes Andrographolide-induced cell death by attenuation of autophagy and augmentation of caspase dependent and independent cell death in HeLa cells. PLoS ONE.

[37] Manigandan K., Manimaran D., Jayaraj R.L., Elangovan N., Dhivya V., Kaphle A. (2015). Taxifolin curbs NF-κB-mediated Wnt/β-catenin signaling via up-regulating Nrf2 pathway in experimental colon carcinogenesis. Biochimie.

